# Evaluation of an authorized nurse immunizer led opportunistic patient influenza and COVID-19 vaccination program under the RE-AIM framework

**DOI:** 10.1093/pubmed/fdaf049

**Published:** 2025-05-11

**Authors:** Sarah Davies, Kathryn Taylor, Donna Moore

**Affiliations:** Public Health Unit, Central Coast Local Health District, 77a Holden Street, Gosford 2250, NSW, Australia; Public Health Unit, Central Coast Local Health District, 77a Holden Street, Gosford 2250, NSW, Australia; School of Medicine and Public Health, College of Health, Medicine and Wellbeing, The University of Newcastle, University Dr, Callaghan 2308, NSW, Australia; Public Health Unit, Central Coast Local Health District, 77a Holden Street, Gosford 2250, NSW, Australia

**Keywords:** immunisation, models, public health

## Abstract

**Background:**

Influenza and COVID-19 are significant vaccine-preventable causes of morbidity, mortality, and healthcare costs. Hospital-based, nurse-led models can increase vaccine uptake, yet few target adults. This study evaluates the implementation of an opportunistic patient influenza and COVID-19 vaccination program in the Central Coast Local Health District (CCLHD), led by Authorized Nurse Immunizers (ANIs).

**Methods:**

Evaluation of the ANI-led program was conducted using the RE-AIM (reach, effectiveness, adoption, implementation, and maintenance) framework. The 10-week program involved screening patients for vaccine eligibility, offering vaccinations and collecting quantitative data and qualitative feedback across the five RE-AIM domains.

**Results:**

Of 849 patients screened alongside service encounters, 398 were vaccinated with 76% of eligible patients consenting to flu and 59% to COVID-19 vaccination. Inpatients had lower vaccination rates on admission compared to the general population and higher rates after program contact. The program was well received by patients and staff and adopted across various CCLHD settings, effectively addressing community access barriers.

**Conclusions:**

Opportunistic vaccination using an ANI-led model is an effective strategy to improve vaccination coverage among higher-risk patients. This evaluation demonstrates the benefits of a dedicated nurse immunizer workforce and suggests potential for broader adoption in similar healthcare settings to improve public health outcomes.

## Introduction

Influenza and COVID-19 infections are significant, yet vaccine-preventable causes of morbidity, mortality and health system costs. Sentinel network data in Australia estimated influenza vaccination averted 50% of hospital admissions in the elderly, 41% in non-elderly adults and 25% in children in a typical season.^1^^,^^2^ Similarly, studies of COVID-19 vaccination effectiveness have shown risk of hospitalisation or death to be reduced by 40%–66%.^3^^,^^4^ In Australia annual influenza vaccination is recommended for people aged 6 months and over and publicly funded for those 6 months to 5 years, 65 years and over, and those at higher risk. Similarly, COVID-19 vaccines are publicly funded in Australia for anyone eligible.

In Australia, most vaccinations are delivered in primary care (in 2023, 61% in general practice settings and 18% in pharmacies).^5^ However hospital-based programs have demonstrated opportunities to increase uptake for at-risk children and adults.^6–12^ Further, programs using authority of healthcare workers to administer vaccinations to eligible patients without physician supervision have been shown to be most effective in increasing inpatient coverage.^9^ Nevertheless, the extent to which vaccination is embedded into routine patient care in Australian hospitals varies widely and is dependent on local drivers.

In 2020, Central Coast Local Health District (CCLHD) successfully piloted an opportunistic inpatient influenza vaccination program led by Authorized Nurse Immunizers (ANIs). ANIs are registered nurses who have completed an approved program of study allowing them to prepare and administer a range of vaccines without a medication order from a doctor or nurse practitioner.^13^ Continuation of the pilot was disrupted by the COVID-19 response but in 2023, a new program based on the 2020 model with addition of COVID-19 vaccination was launched, incorporating an evaluation component. This study presents the program evaluation under the Reach, Effectiveness, Adoption, Implementation, and Maintenance (RE-AIM) framework. RE-AIM has been widely used across fields of public health, behavioural science and implementation science for more than 20 years.^14^

## Methods

### Program setting

New South Wales (NSW) state in Australia has 15 Local Health Districts (LHDs) responsible for health service delivery. Central Coast LHD (CCLHD) serves a peri-urban population of 350 000 north of the state capital, Sydney. The Central Coast population is older, has higher rates of many chronic diseases and experiences greater socioeconomic disadvantage compared to the rest of NSW, including suburbs among the most disadvantaged in Australia.^15–17^ Central Coast has lower levels of access to General Practitioner (GP) services compared to national benchmarks^18^ with those in areas of socio-economic disadvantage more likely to delay or not use GP services due to cost.^19^

CCLHD employs 7900 staff, operating three hospitals with a bed capacity around 1080, and eight community health centres. It offers free community clinics for immunisations funded under the National Immunisation Program (NIP) for children 0–5 years and for adolescents in schools.

### Program delivery

Vaccinations were offered by ANIs on average 3 days per week over 10 weeks from April to June 2023 in all three CCLHD hospitals and selected outpatient settings. In Australia the peak flu season is generally June to September and flu vaccination is encouraged from April onwards. The 10-week period aligned with CCLHDs winter vaccination campaign. An ANI Team Lead worked 3 days a week, coordinating program visits and ANI staff. Generally, two ANIs worked with patients with a third team member rostered when patient numbers were higher. CCLHD’s Immunisation Coordinator provided clinical support.

Program information was communicated to CCLHD staff, who could register interest for patients to participate. The program also proactively contacted Nurse Unit Managers (NUMs) in priority areas such as geriatric & aged care, renal, rehabilitation, respiratory, cardiology, mental health and drug & alcohol services. These areas were prioritized due to the increased risk from respiratory infections to these patients. The program did not exclude any service that expressed interest in accessing vaccinations for patients.

### Program eligibility criteria

Patient lists were generally available before team visits to pre-screen on the Australian Immunisation Register (AIR) for eligibility and further patient screening took place with NUMs and electronic medical records. Family members, GPs and/or the treating team were contacted as appropriate. If any doubts as to eligibility, the team did not proceed. Vaccinations were recorded in the electronic medical record and to the AIR. Patients were eligible:

- if there were no medical contraindications
- if able to provide informed valid consent (patient or guardian)
- for influenza vaccination if not already vaccinated with a 2023 seasonal vaccine.
- for a COVID-19 bivalent booster if
  - over 18 years of age
  - a primary course had been completed and
  - they had not received COVID-19 vaccination or had COVID-19 infection within the previous 6 months.

This was in accordance with national recommendations at the time. COVID-19 primary course vaccination was not offered, as the recommended vaccine during this period was not stocked by CCLHD.

### Data collection

Demographic and eligibility information was collected on all screened patients from patient records, and the number of influenza and COVID-19 vaccinations in the previous three years from AIR as an indicator of attitudes towards vaccination. Hospital pharmacy records provided the number of clinician-prescribed influenza vaccines delivered outside the ANI-led program.

From program week five, patients could complete a voluntary survey immediately after interacting with the ANI team. This survey asked questions about intention to be vaccinated, whether the ANI team adequately addressed any questions or concerns and reasons for consenting or declining vaccination. Post-program, all CCLHD NUMs were invited to complete a survey on their program experience which included questions on perceived responsibility and confidence with influenza and COVID-19 vaccination for patients, as well as feedback on the program model. Both surveys used REDCap version 13.0.0 with consent given during survey commencement. In addition, a debrief of program staff collected feedback and future recommendations.

The program evaluation was ethics reviewed and authorized by the Central Coast Local Health District Research Office (ref: 0423-036C).

### Analysis

R version 4.3.1 was used for statistical analysis. Wilcoxon Rank Sum test and Fisher’s Exact test were used to compare differences between groups. Responses to survey questions were summarized by frequencies and percentages. Open-ended question responses were grouped by common themes.

## Results

### Reach

**Figure 1:**
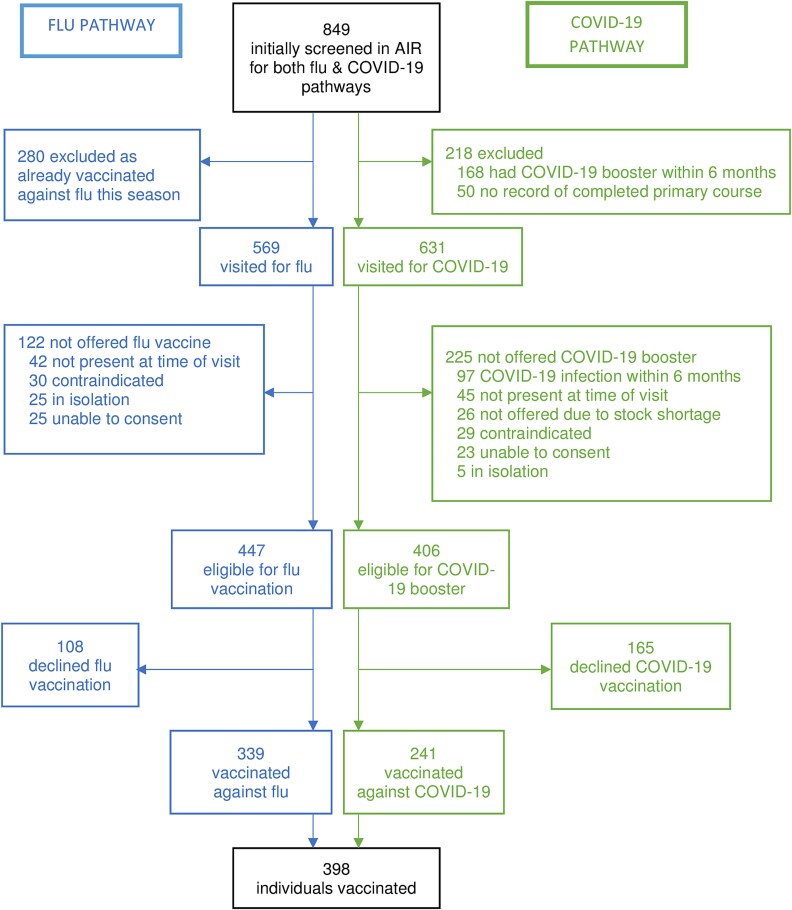
Flowchart summarising participation.

849 patients were screened, with 398 individuals vaccinated against influenza and/or COVID-19. Most were inpatients (678, 79.9%), the remainder attending renal, mental health, drug and alcohol, antenatal diabetes outpatient clinics or Aboriginal health services. After excluding ineligible patients, 447 were offered influenza vaccination, with 339 (75.8%) consenting. 406 were offered COVID-19 vaccination, with 241 (59.4%) consenting (Fig. 1). There was no significant difference in sex or age between those consenting and declining influenza vaccination. For COVID-19 vaccination the average age of those consenting was slightly higher than those declining. For both influenza and COVID-19 vaccination, those consenting were more likely to have been fully vaccinated in the previous three years compared to those declining vaccination (Table 1).

**Table 1:** Demographics of patients screened, surveyed and vaccine eligible.

Patients	Screened (n = 849)	Surveyed (n = 135)	P-value		
Number females	418 (49.2%)	65 (48.1%)	0.853		
Median age (IQR)	77 (24)	76 (17)	0.909		
Number inpatients	654 (77.0%)	119 (88.1%)	**0.003**		
Median length of stay for admitted patients in days (IQR)	26 (46)	32 (48.5)	0.782		
Number vaccinated for flu every year in previous 3 years^a^	384 (45.2%)	62 (45.9%)	0.926		
Number with 4 or more COVID-19 vaccinations in previous 3 years^a^	455 (53.6%)	75 (55.6%)	0.711		
Consented to flu vaccination (% of those eligible)	339 (75.8%)	74 (82.2%)	0.218		
Consented to COVID-19 vaccination (% of those eligible)	241 (59.4%)	81 (77.1%)	**0.0007**		
*Flu vaccination*	*Eligible (n = 447)*	*Consented (n = 339)*	*Declined (n = 108)*	*P value*	*OR (95% CI)*
Number females	214 (47.9%)	154 (45.4%)	60 (55.5%)	0.0768	
Median age (IQR)	71 (30)	71 (31)	69 (28)	0.973	
Number vaccinated for flu every year in previous 3 years^#^	147 (32.9%)	129 (38.1%)	18 (16.7%)	**3.2** × **10**^**−5**^	**3.06 (1.74; 5.66)**
*COVID-19 vaccination*	*Eligible (n = 406)*	*Consented (n = 241)*	*Declined (n = 165)*	*P value*	
Number females	191 (47.0%)	106 (44.0%)	85 (51.5%)	0.157	
Median age (IQR)	70 (30)	71 (26)	64 (30)	**0.0207**	
Number with 4 or more COVID-19 vaccinations in previous 3 years^a^	183 (45.1%)	135 (56.0%)	48 (29.1%)	**1.1 × 10** ^**−7**^	**3.10 (2.00; 4.85)**

a Based on Australian Immunisation Register records which may be incomplete

Patient survey respondents were representative of the screened group in terms of age, sex, and vaccination history. A higher proportion of inpatients participated and eligible patients who completed the survey were more likely to consent to COVID-19 vaccination (Table 1).

Survey results indicated low levels of vaccine hesitancy. Of 135 respondents, 45 (33.3%) had already been vaccinated against influenza. Of the remainder, 74 (82.2%) consented and of those, 66 (89.2%) stated they were likely to seek influenza vaccination in the current year. A small number (7, 7.8%) who stated they were unlikely to be vaccinated, nevertheless consented. Main reasons for consent were convenience, worry about getting the flu and always getting the flu shot.

For COVID-19 vaccination, 30 (22.2%) survey respondents were ineligible for a booster. Of the remainder, 81 (77.1%) consented and of those, 75 (92.6%) stated they were likely to seek a COVID-19 booster in the current year. Of patients who stated they were unlikely to seek a booster, 15 (14.3%) nevertheless consented to vaccination. As with influenza, main reasons for consenting were convenience and worry about getting COVID-19 (Table 2).

**Table 2:** Patient survey responses—reasons for consenting and declining vaccinations.

Reasons given for consenting to vaccination (multiple answers possible)	Influenza (n = 71)	COVID-19 (n = 78)
Convenience of offer in hospital/clinic	51 (71.8%)	57 (73.1%)
I always get a flu shot	37 (52.1%)	-
I’m worried about getting the flu/COVID-19	25 (35.2%)	33 (42.3%)
Recommended by a health professional	12 (16.9%)	20 (25.6%)
To protect those around me	5 (7.0%)	11 (14.1%)
Recommended by a friend or family member	1 (1.4%)	1 (1.3%)
*Reasons given for declining vaccination* *(multiple answers possible)*	*Influenza* *(n = 16)*	*COVID-19* *(n = 24)*
I’m worried about side effects	7 (43.8%)	11 (45.8%)
I’m too unwell	7 (43.8%)	5 (20.8%)
Don’t like needles	1 (6.3%)	0
Don’t think I need it	0	10 (41.7%)
I’ve previously been told not to by a health professional	0	1 (4.2%)
I think it’s not effective in preventing flu/COVID-19	0	1 (4.2%)
I think vaccinations are dangerous	0	1 (4.2%)

The most consistent theme in survey comments related to access such as:

Patient A: ‘I don’t have any transport so it’s tricky to get to a GP. I would have had to try and organise it myself but that would take time.’

Patient B: ‘Probably wouldn’t have got to the chemist otherwise to get it, having trouble with my leg.’

Reasons for declining vaccination showed some difference in attitudes between influenza and COVID-19 (Table 2). Feeling too unwell and being worried about side-effects featured for both; however, only COVID-19 elicited the response ‘I don’t think I need it’.

### Effectiveness

Effectiveness was defined as the program’s ability to increase vaccination coverage. In the inpatient setting, the ANI-led model administered 238 influenza vaccines in addition to 13 clinician-prescribed vaccines. This compared to 133 clinician-prescribed vaccines in 2022, an 89% increase. The ANI-led model was the only avenue for inpatient COVID-19 vaccination in CCLHD facilities in 2023; no service was available in 2022.

Comparison of influenza vaccination uptake between the program’s cohort and the Central Coast population aged 65 years and over showed consistently lower vaccination rates; 21.4% compared to 33.1% at end April and 33.1% compared to 65.3% at end June. Access to the program for this at-risk cohort led to coverage rates exceeding the wider Central Coast population by 31.2% at end April and by 7.8% at end June. No comparable data for COVID-19 is available.

In the outpatient/community clinic setting, likely improvements in flu vaccine coverage were also seen (Table 3). Only 22% of outpatients who consented to flu vaccination had been consistently vaccinated in the previous 3 years, with inpatients 2.9 times more likely than outpatients to have been regularly vaccinated. Inpatients and outpatients who consented to COVID-19 vaccination were similarly up-to-date.

**Table 3:** Comparison of recent vaccination history for inpatients and outpatients.

	Consented Inpatients (Flu: n = 238) (COVID-19: n = 158)	Consented Outpatients (Flu: n = 101) (COVID-19: n = 83)	P value	OR (95% CI)
Number vaccinated for flu every year of the previous 3 years^a^	107 (45%)	22 (22%)	5.0 × 10^−5^	2.92 (1.67; 5.27)
Number with 4 or more COVID-19 vaccinations in the previous 3 years^a^	94 (59%)	41 (49%)	0.172	1.50 (0.85; 2.66)

a Based on Australian Immunisation Register records which may be incomplete

### Adoption

ANIs visited 26 different hospital wards, seven outpatient clinics and four community centres. The program team initiated most visits, with five NUMs proactively registering interest. As the program progressed more referrals were received for specific patients. Two NUMs working with drug and alcohol services were initially unsure how the program would align, a pre-visit from the team addressed concerns and successful clinics were run.

Fifteen NUMs responded to the post-program survey which explored attitudes to inpatient vaccination and the ANI-led model. 12 (80.0%) respondents were aware of the program and seven (46.7%) had contact with the team in their ward/clinic. 70% considered patient immunisation part of their care responsibilities and 67% felt it was easy to keep up-to-date with vaccination recommendations. 70% stated their interaction with the program gave more confidence to discuss immunisation with patients and 80% had greater awareness of the ANI role. All NUMs who interacted with the program agreed it was an effective strategy to reach patients. Survey comments highlighted good communication and coordination and value of the service to patients, contributing to high levels of adoption.

NUM A: ‘Fantastic service, very easy to contact and refer to.’

NUM B: ‘This program has been extremely valuable. Long term patients have no opportunity to attend outpatient clinics and are high risk.’

NUM C: ‘Good communication…Limited interruption on ward. Straight forward process.’

### Implementation

Few program adaptations were made during implementation, with planning in 2023 informed by lessons from the 2020 pilot. Key changes were addition of staff resources to support coordination, planning and data analysis. One adaptation made was using hospital patient flow software to streamline initial patient screening.

From CCLHD’s perspective, program costs were mainly ANI staff hours at ~AUD61.30 per vaccine administered. Vaccines were provided free of charge through State and National Programs. Equipment and consumables were sourced from existing stock without program cost, however if commencing de novo ~AUD6000 would be required for equipment initially and AUD200/year for consumables.

### Maintenance—setting level

The program is designed to be seasonal with future intention to embed within regular clinical services. Feedback collected provided recommendations to adapt/improve next iterations including additional preparation time, running for 12 weeks and maintaining team leader and administrative support roles.

## Discussion

### Main finding of this study

Our evaluation of this novel nurse immunizer-led model in an Australian context demonstrates positive outcomes adaptable to other settings. Our study found 66% of eligible patients overall and 61% of those aged 65 and over were not vaccinated against influenza before admission. This is substantially higher than previous Australian studies of hospitalized patients and compared to the general population. Similarly, 69% of eligible patients were not up-to-date with COVID-19 boosters demonstrating opportunities for improvement. Most eligible patients in our study received vaccinations when offered. Offering free opportunistic immunisations to patients at healthcare touchpoints effectively addressed community access barriers and increased coverage within higher-risk groups.

### What is already known on this topic

While benefits of opportunistic vaccination initiatives are well-established,^7^^,^^9^^,^^20^^,^^21^ numerous barriers hinder integration into clinical care making reported inpatient vaccination rare. For example, in Melbourne tertiary hospitals, 23–24% of eligible patients (majority over 65) were found to be unvaccinated against influenza before admission.^11^^,^^12^ In addition, only one of 46 inpatients were subsequently vaccinated against influenza in one study^12^ and none of 3864 in another.^11^ Similarly, a cross-sectional survey of 1292 Australian adults 45 years and over found 28.3% who attended hospital were not immunized for influenza in the previous year and only seven reported receiving an influenza vaccine at hospital.^8^

COVID-19 vaccine uptake is less straightforward for comparison given recent infection affected booster recommendations in Australia in 2023. Patient surveys earlier in the pandemic demonstrated lower coverage in patients presenting to hospital in Australia compared to the general population.^10^^,^^22^^,^^23^

Nurse-driven interventions have been effective in increasing vaccination coverage across a range of health-care settings.^9^ Many of these interventions employ nurse-led Standing Order Protocols, similar to the ANI-led model in that they broaden responsibility for vaccination beyond prescribing physicians, however, no other example of a dedicated team of nurse immunizers targeting adult inpatients was found in the literature. A similar model was found for opportunistic immunisation of paediatric inpatients, successfully tested in a 5-month pilot in Melbourne in 2013/2014^7^ which continues to operate. Its most recently published data shows around 100 inpatients per month identified as due or overdue for NIP immunisations and between 50%–70% of these brought up-to-date each month.^24^

Inpatient vaccination programs have possible net savings through avoiding or reducing length of admissions. Estimates of potential savings were outside the scope of this evaluation but a study in a Melbourne hospital, estimated potential net savings of vaccinating general medical inpatients against influenza between AUD192 to AUD1489 per patient per year.^25^

### What this study adds

Experience from this program adds to the evidence that a dedicated nurse-led service targeting higher risk patients will improve vaccination coverage and increase equitable access.

Lower initial vaccination coverage was expected in our cohort, given this was checked during the early flu season, and after admission during sometimes prolonged stays (approximately one in four stayed 60 days or more). However, the difference with other published studies was marked. These results, along with comparison to the wider Central Coast population, indicate the higher-risk groups targeted by the program are likely poorly covered.

Within CCLHD, outside the ANI-led model, no consistent system exists for recording vaccination status and triggering an offer of indicated immunisations. Given missed opportunities for vaccination described in the literature, this appears common, with findings of as low as 2.3% of hospitalized patients having vaccination status recorded.^11^ The proportion of eligible patients consenting to vaccination in our study (76% for influenza, 59% for COVID-19) is similar to the willingness to be vaccinated in hospital reported in recent Australian surveys for influenza and pneumococcal (72.2%)^8^ and for COVID-19 (54%).^10^ This further supports the potential of this model to bridge access barriers and improve coverage. The ANI-led model was also readily adaptable to a wide range of inpatient, outpatient and community settings, offering greater flexibility than programs requiring medical oversight. Moreover, the NUM survey showed improved awareness of the nurse immunizer role and more confidence to discuss immunisation with patients which may assist in promoting inpatient vaccination in the future.

Higher flu vaccination uptake compared to COVID-19 likely reflects differing attitudes and experiences with these vaccines, with survey respondents more likely to state they didn’t need a COVID-19 booster compared to the flu shot. Our program’s patient survey was based on the instrument used in Australian Department of Health research in 2017 and 2021 on community attitudes to influenza vaccination. These surveys all found similar results on reasons for flu vaccination uptake with ‘always getting a flu shot’, ‘being worried about/protecting oneself from infection’ and ‘having the flu vaccine recommended by a health professional’ among top responses. Our patient survey results differed however, with respect to access. The national survey found vaccine access perceived to be ‘very easy’.^26^ Our survey comments revealed physical mobility, illness and/or transport limitations were often significant barriers to patients and convenience was the most important reason for vaccination consent. It is worth noting that for patients with long hospital stays, inpatient vaccination may be their only option. In addition, our analysis of recent vaccination histories showed higher-risk outpatients were less likely to be vaccinated against flu than inpatients. This finding may be a result of targeting outpatient clinics serving more vulnerable groups.

### Limitations of this study

Key limitations of the evaluation were the pilot nature of the model, and the absence of a formally defined comparison group. The evaluation was timed to align with the winter flu vaccination campaign, however, increases in COVID-19 infections in Australia are observed every 3 to 4 months. There is also less historical data for comparison with COVID-19 than for influenza. Additionally, no other strategies for improving vaccination coverage were tested for comparison and a formal economic evaluation was not undertaken. There may also be limitations in the generalisability of findings, particularly outside the Australian context.

## Conclusions

Opportunistic vaccination using an ANI-led model is an effective strategy to improve vaccination coverage among higher-risk patients. Evaluation of this program demonstrated the benefits of a dedicated nurse immunizer workforce and highlighted the importance of addressing access barriers to vaccination. The successful implementation suggests potential for broader adoption in similar healthcare settings to improve public health outcomes.

## Supplementary Material

Patient_survey_questions_fdaf049

Staff_survey_questions_fdaf049

## Notes

**Sarah Davies,** Epidemiologist

**Kathryn Taylor,** Conjoint Lecturer, Director of Public Health

**Donna Moore,** Immunisation Coordinator
