# Supplementary material for: Evaluation of an authorized nurse immunizer led opportunistic patient influenza and COVID-19 vaccination program under the RE-AIM framework
Source: J Public Health (Oxf). 2025 May 11;47(3):e391–9. doi: 10.1093/pubmed/fdaf049 (PMC12395946; doi:10.1093/pubmed/fdaf049)
Supplement: Patient_survey_questions_fdaf049 [file patient_survey_questions_fdaf049.docx]

**Patient Survey Questions**

**Name: ________________________________**

**DOB: ________________________________**

1. Were you vaccinated today for  Flu  COVID-19  Neither Flu nor COVID-19
2. Before coming into the hospital/clinic, how likely were you to get a **flu** shot this year?

Very unlikely Unlikely Neutral Likely Very likely

1. Before coming into the hospital/clinic, how likely were you to get a **COVID-19** shot this year?

Very unlikely Unlikely Neutral Likely Very likely

1. Any questions or concerns you had about getting vaccinated in the hospital/clinic were listened to

Strongly agree Agree Neither agree Disagree Strongly disagree

nor disagree

1. What were the main reasons you decided to get the **flu** shot while in the hospital/clinic? (show question only if Flu selected in question 1)

I always get a flu shot

Recommended by a health professional

(If Recommended by a health professional is selected, show the options)

GP

Hospital doctor

Nurse immuniser

Other _____________________________

I’m worried about getting the flu

Recommended by a friend or family member

To protect those around me

Convenience of being offered the flu shot in hospital

Other ___________________

1. What were the main reasons you decided to get the **COVID-19** shot while in the hospital/clinic? (show question only if COVID-19 selected in question 1)

Recommended by a health professional

(If Recommended by a health professional is selected, show the options)

GP

Hospital doctor

Nurse immuniser

Other _____________________________

I’m worried about getting COVID-19

Recommended by a friend or family member

To protect those around me

Convenience of being offered a COVID-19 shot in hospital

Other ___________________

1. What were the main reasons you decided not to get the **flu** shot while you were in the hospital/clinic? (show question only if Flu is not selected or Neither Flu nor COVID-19 is selected in question 1)

Already had the flu shot this year

Don’t think I need it

I don’t like needles

I think it’s not very effective in preventing the flu

I’m worried about side effects

I think vaccinations are dangerous

I’m too unwell

I think illness from the flu isn’t that bad

I’ve previously been told not to by a health professional

My friends or family don’t think I should

Other __________________________

1. What were the main reasons you decided not to get the **COVID-19** shot while you were in the hospital/clinic? (show question only if COVID-19 is not selected or Neither Flu nor COVID-19 is selected in question 1)

I have had COVID-19 or a COVID-19 booster in the last 6 months

Don’t think I need it

I don’t like needles

I think it’s not very effective in preventing COVID-19

I’m worried about side effects

I think vaccinations are dangerous

I’m too unwell

I think illness from COVID-19 isn’t that bad

I’ve previously been told not to by a health professional

My friends or family don’t think I should

Other __________________________

1. Do you have any feedback on the experience of being offered vaccination in hospital?
