# Supplementary material for: Evaluation of an authorized nurse immunizer led opportunistic patient influenza and COVID-19 vaccination program under the RE-AIM framework
Source: J Public Health (Oxf). 2025 May 11;47(3):e391–9. doi: 10.1093/pubmed/fdaf049 (PMC12395946; doi:10.1093/pubmed/fdaf049)
Supplement: Staff_survey_questions_fdaf049 [file staff_survey_questions_fdaf049.docx]

**Staff Survey Questions**

**Name: (Optional) ________________________________**

**Position: ________________________________**

1. Does the issue of immunisation ever come up during your conversations with medically at-risk patients?  Yes  No
2. If Yes, who usually initiates the conversation?  I do  The patient

Please indicate your response to the following statements:

1. I am confident in my knowledge of the current **influenza** immunisation recommendations and feel comfortable addressing patient questions

Strongly agree Agree Neither agree Disagree Strongly disagree

nor disagree

1. I consider **influenza** risk for my patients and recommend immunisation where indicated

Always Often Sometimes Rarely Never

1. I am confident in my knowledge of the current **COVID-19** immunisation recommendations and feel comfortable addressing patient questions

Strongly agree Agree Neither agree Disagree Strongly disagree

nor disagree

1. I consider **COVID-19** risk for my patients and recommend immunisation where indicated

Always Often Sometimes Rarely Never

1. The Ward Off Winter! Program offering opportunistic immunisation for flu and COVID-19 is an effective strategy to reach at-risk patients

Strongly agree Agree Neither agree Disagree Strongly disagree

nor disagree

1. Through my interaction with the Ward Off Winter! Program I became more aware that my patients can be immunised against flu and COVID-19

Strongly agree Agree Neither agree Disagree Strongly disagree

nor disagree

1. Through my interaction with the Ward Off Winter! Program I became more aware of the role of Authorised Nurse Immunisers

Strongly agree Agree Neither agree Disagree Strongly disagree

nor disagree

1. Do you have any feedback on the operation of the Ward Off Winter! Program? What worked well, what could be improved, should it continue in future years?
2. Would you be willing to be contacted to further discuss any feedback you have given on the program?

Yes  No
